# Understanding the interface between clinical and laboratory staff

**DOI:** 10.4102/ajlm.v3i1.127

**Published:** 2014-07-24

**Authors:** Ankie van den Broek, Coosje J. Tuijn, Lisette van ’t Klooster, Elizabeth Msoka, Marion Sumari-de Boer, Jaffu Chilongola, Linda Oskam

**Affiliations:** 1 Royal Tropical Institute (KIT) Health, the Netherlands; 2 Royal Tropical Institute (KIT), Biomedical Research, the Netherlands; 3 Kilimanjaro Clinical Research Institute (KCRI), Tanzania; 4 Kilimanjaro Christian Medical University College, Tumaini University Makumira

## Abstract

**Background:**

The interface between clinicians and laboratory staff is where the two meet and work together to provide quality care to their clients (patients). Effectiveness of the interface depends on the way the two groups of professionals relate to and communicate with each other. The number and type of tests requested and the use of the test results for clinical decision making can be influenced by the interface between clinicians and laboratory staff. A model to understand the factors and dynamics around the interface is lacking.

**Objectives:**

To propose a new conceptual model to gain insight and analyse factors that influence the laboratory–clinical staff interface.

**Methods:**

To develop the conceptual model, a literature study was performed, regulatory guidelines and standards for laboratories were analysed and discussions were held with experts on the topic.

**Result:**

A conceptual model and analytical framework provided good guidance in understanding and assessing the organisational and personal factors shaping the interface. The model was based on three elements: (1) the three phases of communication (pre-analytical, analytical and post-analytical); (2) the organisational and personal factors of interaction; and (3) the socio-political, economic and cultural context in which clinicians and laboratory staff operate.

**Conclusion:**

Assessment of the interface between clinicians and laboratory workers can be performed in a systematic way. Applying this model will provide information to managers of health institutions and heads of laboratories and clinical departments about what happens when clinicians and laboratory staff interact, thus aiding them in designing strategies to improve this interface.

## Introduction

Diagnostic tests requested by clinicians are performed by laboratory staff and provide clinicians and patients with the test results that are required for clinical decision making. The contribution of laboratory services to clinical decision making not only depends on the performance of the laboratory itself, but also on the behaviour of clinicians with regard to requesting tests and using the results.^1^ Request behaviour is influenced by the interactions between these two health cadres.^2^ A study by Bridges et al.^3^ on interprofessional collaboration highlighted factors that shape the interface, including responsibility, accountability, coordination, communication, cooperation, assertiveness, autonomy, mutual trust and respect. In low-income countries, little research has been performed to understand elements that shape the quality of the interface between clinicians and laboratory workers and their influence on the quality of care. Carter et al.^4^ mention the lack of communication between clinicians and laboratory services, giving various reasons for this from the perspective of the clinicians and the laboratory staff. According to this article, clinicians are not accustomed to teamwork and the laboratory staff may not recognise the clinical importance of their findings, either for clinical decision making or for patient management.^4^

Assessment of the interface between clinicians and laboratory workers will help health workers and their managers understand how factors related to the organisational culture and the personalities of the staff members have an impact on the interface and, therefore, the effectiveness and quality of service delivery.

## Research method and design

**FIGURE 1:**
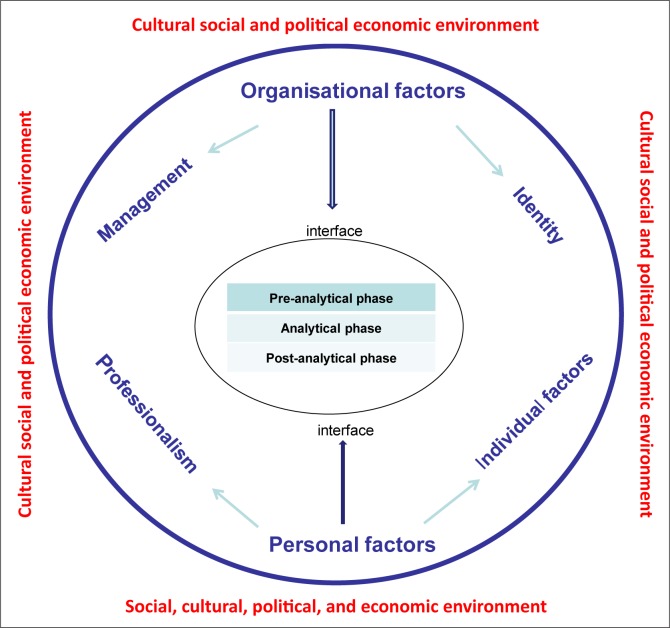
Conceptual model of the various factors that shape the interface between clinical and laboratory staff.

The conceptual model depicted in Figure 1 was developed after completion of a literature search and an analysis of regulatory guidelines and standards for laboratories, followed by a thorough discussion between clinical and laboratory experts within the team. Following this discussion, the study team returned to the literature to search for missing information.

### Literature search

A literature search was performed using a non-systematic approach, as it was known beforehand that minimal peer-reviewed literature would be available and important data could be found in grey literature.

The first literature search (Scopus) yielded 59 relevant peer-reviewed articles, from which 14 were selected as appropriate. Articles were searched for that could provide information about the interactions and interface between laboratory and clinical services by using different search terms referring to the services or to the staff working at these services. The search strategy included the following terms: ‘laboratory services’ AND ‘health systems’; ‘laboratory services’ AND ‘human resources’ OR ‘laboratory personnel’ OR ‘laboratory staff’ OR ‘laboratory workforce’; ‘laboratory services’ AND (‘role’ OR ‘impact’) AND ‘health care’ OR ‘health services’; ‘laboratory services’ AND (‘physicians’ OR ‘clinician’ OR ‘nurses’ OR ‘health manpower’ OR ‘health personnel’ OR ‘medical staff’ OR ‘nursing staff’ OR ‘patients’) AND (‘consumer satisfaction’ OR ‘satisfaction’ OR ‘dissatisfaction’ OR ‘interaction’ OR ‘opinion’ OR ‘attitude of health personnel’); ‘laboratory services’ AND (‘essential’ OR ‘rational’) AND ‘health care’ OR ‘hospital’ OR ‘hospitals’ OR ‘clinic’ OR ‘clinics’ OR ‘medical centre’ OR ‘medical centres’.

The second search performed focused on peer-reviewed articles and grey literature from 1995 onwards using Google Scholar, ISI Web of Knowledge, PubMed, the Royal Tropical Institute website, Science Direct, Scopus and the World Health Organization website. The following search terms were used: ‘attitude of health personnel’; ‘laboratories/ utilization’; ‘physicians/psychology’; ‘trust’ AND ‘clinician’ OR ‘health workers’; ‘laboratory quality’; ‘national laboratory guideline’; ‘laboratory strengthening declaration’. The term ‘interface’ was not used in the search strategy as it was not expected to increase the number of articles related to the interface between laboratory workforce and clinicians; when both types of health workers are mentioned, all articles concerning this interface appear. Adding the term ‘interface’ would yield the retrieval of articles that discuss the interface between the functioning of the laboratory and computerised systems used in the laboratory.

This search was followed by the snowball literature search method.

### Analysis of guidelines

The analysis of regulatory guidelines and standards for laboratories (International Organization for Standardization [ISO] 15189,^5^ ISO 22869^6^ and ISO 9001;^7^ Clinical and Laboratory Standards Institute [CLSI] GP 26;^8^ Joint Commission International [JCI]^9^) provided information on the proposed daily practices for human resource management and relationship building between the laboratory and clinical departments. The interface between the clinicians and laboratory staff is addressed in these documents. An analysis showed that *maintaining the relations with customers* and *monitoring of the customer satisfaction* (clinicians are considered to be customers of the laboratory) are both included in several of these regulatory guidelines (Table 1).

**TABLE 1:** Issues in ISO 15189, ISO 22869, ISO 9001, CLSI GP 26 and JCI guidelines that refer to the interface between the laboratory and clinical departments.

Maintaining relations with parties outside the laboratory	Monitoring of customer satisfaction / Complaint management
Necessary policies are developed for communication with clinicians who order tests (JCI)	Policy and procedures for the resolution of complaints or other feedback received from clinicians, patients or other parties (ISO 15189 & ISO 22869)
Responsibility for the relationship with any other organisation with which the laboratory may be associated (ISO 15189 & ISO 22869)	
Relate and function effectively (including contractual arrangements if necessary with the healthcare community (ISO 15189 & ISO 22869)	Monitor information relating to customer perception as whether the organisation has met customer requirements (ISO 9001)
Communication and coordination throughout the laboratory and with outside customers (JCI)	
Leaders communicate to laboratory staff and employees the priority of meeting the needs of *clinicians*, patients and other users of laboratory services (JCI)	Ensure that customer requirements are determined and meet with the aim of enhancing customer satisfaction (ISO 9001)
Effective and immediate communication with clinicians when clinicians require emergency tests or when results indicate the need for such communication (JCI)	Assess the satisfaction of its external physician, nurse, referring customers and patients with the quality of its services (CLSI GP 26)
Meetings with the professional staff with the clinical staff regarding the use of laboratory services and for the purpose of consultation on scientific matters (ISO 15189 & ISO 22869)	

The importance of including the interface in regulatory guidelines and standards for laboratories is also recognised by Yao et al.,^10^ who mention the consultation of the client and client satisfaction surveys as being key areas on the road to laboratory accreditation.

Based on the findings from the literature search, intensive discussions were held amongst the clinical and laboratory experts in the study team. It was determined that the quality of the interface is related to the moment when interaction happens, the organisational culture of the health facility and the personalities of the clinicians and the laboratory staff.

### Testing

The conceptual model was tested during a mixed-method field study in four health facilities in Moshi District of Kilimanjaro region, in Tanzania. This field study is described in ‘The interface between clinicians and laboratory staff: a field study in northern Tanzania’ by Tuijn et al.^11^

## Results

### The conceptual model

The conceptual model is based on three elements: (1) the phases during which communication takes place; (2) the organisational and personal factors that influence the interface; and (3) the social, political, cultural and economic context in which the health facility operates.

#### Inner circle: Three phases where communication takes place

Plebani^12^ argues that clinicians and laboratory staff interact during the pre- and post-analytical phases; this concept was taken as being the starting point of the framework. A third phase was added to Plebani’s concept, namely, the analytical phase. Application of Plebani’s concept to the analytical phase refers to communication that takes place during the performance of a test or a range of tests; for example, a laboratory worker may ask for clarification on the sample type, sample volume, or information regarding the patient’s situation in order to understand the test results or to suggest additional tests. Incidentally, the clinician can be present when a test is performed and the outcome can be discussed based on observations by both health cadres (although this does not happen often). In this way, the phases are linked to the time frame in which the clinician (on behalf of the patient) asks for the diagnostic test, waits for the test results and develops a plan after receiving the results. In all three phases, information sharing between clinicians and laboratory staff can take place.

During these phases, a number of activities and interactions take place, as are listed in Table 2. This list of activities is based on the knowledge and experience of clinicians and laboratory scientists in the research team.

**TABLE 2:** Activities and interactions taking place between clinicians and laboratory staff during the pre-analytical, analytical and the post-analytical phases.

Activities	Interactions
**Pre-analytical phase**	Requesting of (correct) tests, sample type (material to examine), checking the availability of tests.
	Clinician sharing (relevant) information with the laboratory staff.
	Collection and transportation of the samples often done by an intermediate health worker (nurse).
	Questions for clarification regarding the tests to be performed or patient information by the laboratory staff.
**Analytical phase**	The clinician is present during the test or co–examines the test (e.g. rapid tests, microscopy).
	There are a range of tests requested by the clinician. Between the separate analytical procedures of various tests, contact concerning any kind of information can be sought such as additional patient information or to discuss the choices for additional tests to be performed or withdrawn.
**Post-analytical phase**	Reporting of results including information for interpretation by the clinician (such as normal ranges).
	Presenting the results to the clinician, often done by an intermediate health worker (nurse).
	Clinician giving feedback on the results to the laboratory.
	Discussions between the clinician and laboratory staff concerning the test results.
	Possible requirement for re-testing or additional tests.

#### Outer circle: Factors influencing relationships and communication

Communication between clinicians and laboratory staff is influenced by: (1) organisational factors, such as the management (rules, guidelines, meetings) and the identity of the organisation; and (2) personal factors (knowledge, attitude, competencies) visualised in the outer circle of the conceptual model. In some cases, no literature could be found to support the opinion that certain factors influence the dynamics in the interface. As explained in the methodology, the factors adopted in the model were based on the experiences of the study coordinators.

#### Organisational factors

**Identity of the organisation:** Identity can influence many aspects in an organisation such as conditions for thinking and learning; possibilities for open communication between management and staff; and communication between health cadres working in various departments of the health facility. For this article, the identity of a health organisation in a low-income country was defined based on its position in the health system (primary, secondary or tertiary level); the ownership of the organisation (public, private – faith-based or secular); and the organisation’s profit status (not-for-profit or corporate). It was assumed that the identity of the organisation has a bearing on the level of knowledge of clinicians and laboratory staff. For example, the level of education and opportunities for continuous professional development are different for staff working in referral hospitals compared with staff working in primary healthcare facilities. Monitoring and supervision of health staff can be organised differently in public and private health facilities.

The identity of an organisation can also relate to the availability of resources, such as equipment, consumables and human resources, as well as to leadership and management styles, thus influencing the motivation of the health staff. It can also influence the personalities and the relationships between people working in an organisation: in faith-based organisations, it is likely that staff have activities in common outside working hours (e.g. church activities) that can influence the communication channels in the health facility.

Although the literature study did not provide information related to the identity of the organisation, this factor was added to the framework as it was assumed that this is an important variable for the interface between different departments in a health institution.

**Style of management:** The management can influence the interface between the clinical and laboratory departments in a health institution in three ways:

1. Provision of rules and guidelines for the requesting of diagnostic tests (selecting the correct test, following correct test requisition methods, being aware of the availability of tests) and reporting of test results. Here. the role of an ‘intermediate health worker’, often a nurse, is important to take into consideration, but was not noted in the literature search. However from the field study it was learned that a nurse is often responsible for taking samples to the laboratory and collecting the test results, as well as transferring them to the clinician.
2. Facilitation of mandatory and voluntary meetings in which clinical and laboratory staff participate and interact.
3. The style of management that influences the performance and motivation of health workers by mechanisms such as availability of job descriptions, supportive supervision of staff, implementation of staff appraisals, provision of opportunities for continuous education and career development and demonstration of appreciation for the staff’s work.

These mechanisms also influence the interaction between different cadres of health workers that need each other’s competencies in order to perform their work.

#### Personal factors

The personal factors that influence the interface are the individual competencies of the health workers, including knowledge, attitude and skills, as well as issues relating to the professionalism and professional education of the clinical and laboratory staff. In the literature, several examples are provided regarding personal factors that influence the laboratory–clinician interface. Table 3 provides an overview of the evidence found in the literature regarding these personal factors.

**TABLE 3:** Evidence found in peer-reviewed and grey literature on personal factors that have an influence on the interface between clinicians and laboratory staff.

Knowledge and skills of clinicians and laboratory staff	Attitude related to procedures performed by clinical and laboratory staff	Professionalism
Unawareness of clinicians of the (possible) consequences of patients’ clinical features on the outcome of the tests leading to insufficient information provision to the laboratory services. The laboratory staff – if aware of this – could request additional information.^4^	Clinicians request tests or collect specimens that need quick analysis without informing the laboratory staff in advance leading to unreliable test results.^4^	The laboratory worker analyses specimens in a scientific manner to create an outcome, the clinician also uses experience and common sense to come to a diagnosis. Insight and understanding in both groups about the different viewpoints may already improve the interaction.^4^
Insufficient filling of request forms can cause confusion in the laboratory, including with patients for a potential medical emergency; this may lead to unacceptable delays in sample taking and testing.^4^	The results of tests are not always used for clinical decision making. This may undermine the motivation of laboratory staff to perform tests accurately. When noticed by clinicians, it leads to reduced confidence in test results.^13^	Hierarchy is strongly embedded in the health sector in lower/middle income countries. The academically-educated clinicians usually work at the management level in health institutions, whilst laboratory staff are answerable to them.^4^
Unawareness of laboratory staff of the effect of the presentation of the test results; clinicians face problems as a result of non-interpretable presentation formats.^14^	Lack of motivation of laboratory staff in peripheral-level laboratories feeling neglected and isolated.^15^	Lack of confidence of clinicians in laboratory results, leads to inappropriate use of test results.^8^ The lack of trust may constrain the laboratory services as inefficient use of tests and their results can lead to higher costs in an already resource-poor settings.^14^
Clinicians regularly cannot distinguish between an outcome that could be correct and an outcome that does not fit the patient’s clinical picture; and may not identify mistakes made by the laboratory.^4^	-	-
Despite test results, clinicians proceed with unnecessary or incorrect treatment.^4,16^ This can also be caused by inadequate knowledge of the national or global policies by clinicians and laboratory services.^17^	-	-
‘Clinicians are not using standard criteria for ordering malaria tests, but base their request behaviour on their own rationality and 20% to 60% of the ordered tests are clinically unnecessary’.^13,18,19^	-	-
Unawareness on the part of clinicians (and laboratory staff) of tests available.^17^	-	-

The individual competencies of clinical and laboratory staff can be insufficient because of lack of updates on national policies and guidelines, lack of supervision and coaching and lack of motivation to understand the impact of inaccuracy on the quality of care. In the field study described by Tuijn et al.,^11^ evidence was found suggesting that lack of competencies can also be a result of a low level of education, especially amongst laboratory workers. Many tasks in the laboratory are performed by laboratory attendants without formal training.

During the field study, it was determined that the needs and wishes of patients could influence clinicians’ test-requesting behaviour or their use of test results. When waiting times at the laboratory were long, clinicians did not ask for a repeat test, as it could be inconvenient for the patient. When a clinician was influenced in his request behaviour, it was classified as a personal factor. However, management can have an impact on personal factors when, through guidelines and supervision, these issues are discussed.

Issues related to professional education and professionalism are: (1) the different professional viewpoints of clinicians and laboratory workers: the laboratory worker is focused on the outcome of the test as the gold standard for treatment whilst the clinician values it as additional information to confirm or complete the clinical diagnosis; (2) the institutionalised professional positions of both cadres in the health system and health facility in which clinicians occupy a higher position in the hierarchy; and (3) trust in the quality of the work of the other health professional: for the clinician to make decisions regarding diagnosis and treatment and for the laboratory worker to make decisions regarding the test procedures and interpretation of results, each needs to rely on the competence of the other.

#### Relationship between organisational factors and personal factors

Organisational factors and personal factors are partly interdependent. Management of the health facility can influence the individual factors and professionalism through attrition and deployment policies, supervisory mechanisms and opportunities for continuous professional education. The management style can also impact staff motivation and the acceptance and ability of the various health professionals to communicate with persons in different positions and different fields of expertise.

The identity of the health facility can also have an influence on the type of job applicants and on personal contacts between staff members (e.g. workers at faith-based hospitals often meet during church activities).

#### Context

In the outer square of the conceptual model is the context in which the health institution operates. Social, cultural, political and economic factors have an impact on the hospital and its health workers. Many low-income countries experience a severe shortage of human resources for health, leading to understaffing or employment of insufficiently-qualified staff in several departments of the hospital.^13,14,15,16,17,18,19,20^ The field study confirmed the shortage of qualified laboratory staff, leading to a situation where staff with less education take on responsibilities that include communicating with highly-educated clinicians.

#### Analytical framework

We developed an analytical framework that serves as a guide when assessing the interface between laboratory and clinical staff. In this framework, all the factors and activities that influence the dynamics around the interface in the three phases are brought together. This analytical framework was used and is explained in more detail in the field study, ‘The interface between clinicians and laboratory staff: a field study in northern Tanzania’, by Tuijn et al.^11^

## Discussion

This model is new and was developed for health services in low-income countries, which face challenges with regard to service delivery in resource-constrained settings. The conceptual model and the framework provide an overview of factors that determine the interface between clinicians and laboratory workers. All of the factors that provided the basis for this conceptual model informed the study team about the complexity of this interface, but also showed that a well-functioning interface can contribute to quality of care. In low-income countries, little attention is given to this interface, even though the high workload for many of the health workers requires the efficient use of all services and thus efficient cooperation between services to provide quality care. By creating awareness in both groups and improving the interface between the clinicians and laboratory staff, use of laboratory services can be optimised, enabling clinicians to make better diagnoses and treatment plans for their patients.

In developing this model, it was determined that the identified peer-reviewed articles do not provide information regarding all factors that influence an effective interface; for example, the interdepartmental management was hardly discussed. However, the importance of interdepartmental management is partly addressed in the reviewed regulatory standards for laboratories, which mention development and/or monitoring of relationships with clients, including physicians, as well as policies regarding interdepartmental meetings.

Several factors which were included in the model were not mentioned in either peer-reviewed articles or regulatory standards, for example, general personal factors such as age and gender, and cultural factors such as hierarchy in relationships or family ties, but it was assumed that these factors influence staff attitudes and they were thus included in the framework. The pilot field study provided indications that these factors influence the communication between clinicians and laboratory staff, but the study was too small to make firm conclusions about this issue.

The complexity of the interface, which is influenced by organisational and personal factors as well as the health facility’s context, calls for a holistic analysis involving all stakeholders (clinicians, laboratory staff, intermediate health workers such as nurses, the management and patients or clients). This first field study has demonstrated the robustness of this conceptual model. It enables analysis of the factors that shape an effective interface. The plan is to perform an assessment of the interface with a sample of 20 health facilities to increase the evidence on these factors. Outcomes of such a study may motivate managers of health institutions and heads of laboratories and clinical departments to invest in analysis of interdepartmental interaction so that, based on their findings, they can design strategies to improve the interface in their settings.

## Conclusion

A new conceptual model has been developed to assess the interface between laboratory and clinical staff in low income countries.
